# Spinal Cord Stimulation and Treatment of Peripheral or Central Neuropathic Pain: Mechanisms and Clinical Application

**DOI:** 10.1155/2021/5607898

**Published:** 2021-10-21

**Authors:** Liting Sun, Changgeng Peng, Elbert Joosten, Chi Wai Cheung, Fei Tan, Wencheng Jiang, Xiafeng Shen

**Affiliations:** ^1^The First Rehabilitation Hospital of Shanghai, School of Medicine, Tongji University, Shanghai, China; ^2^Advanced Institute of Translational Medicine, Tongji University, Shanghai, China; ^3^Department of Anesthesiology and Pain Management, Maastricht University Medical Center, Maastricht, Netherlands; ^4^Laboratory and Clinical Research Institute for Pain, Department of Anaesthesiology, University of Hong Kong, HKSAR, China; ^5^Shanghai Skin Disease Hospital, School of Medicine, Tongji University, Shanghai, China

## Abstract

Spinal cord stimulation (SCS) as an evidence-based interventional treatment has been used and approved for clinical use in a variety of pathological states including peripheral neuropathic pain; however, until now, it has not been used for the treatment of spinal cord injury- (SCI-) induced central neuropathic pain. This paper reviews the underlying mechanisms of SCS-induced analgesia and its clinical application in the management of peripheral and central neuropathic pain. Evidence from recent research publications indicates that nociceptive processing at peripheral and central sensory systems is thought to be modulated by SCS through (i) inhibition of the ascending nociceptive transmission by the release of analgesic neurotransmitters such as GABA and endocannabinoids at the spinal dorsal horn; (ii) facilitation of the descending inhibition by release of noradrenalin, dopamine, and serotonin acting on their receptors in the spinal cord; and (iii) activation of a variety of supraspinal brain areas related to pain perception and emotion. These insights into the mechanisms have resulted in the clinically approved use of SCS in peripheral neuropathic pain states like Complex Regional Pain Syndrome (CRPS) and Failed Back Surgery Syndrome (FBSS). However, the mechanisms underlying SCS-induced pain relief in central neuropathic pain are only partly understood, and more research is needed before this therapy can be implemented in SCI patients with central neuropathic pain.

## 1. Introduction

Electrostimulation for pain therapy emerged in the convergence of Pacemaker technology, the “Gate control” theory of pain, and pioneering clinical trials from 1950s to 1960s [1, 2]. According to this theory, the activation of low-threshold nonnociceptive fibers closes the gate of the nociceptive signal input through the activation of inhibitory neurons in the spinal cord to suppress pain [1]. SCS is a form of electrotherapy by implanting electrodes into the epidural space in the spinal cord and stimulating the dorsal column to modulate neural function. The first-generation of SCS devices was comprised of two main components: an electrode and a pulse generator. The original electrode was a design based on Torresani et al.'s cardiac pacemaker that incorporated twisted platinum tinsel wire in a Dacron filament matrix [3]. SCS was first successfully used in 1967 by Shealy et al. for the treatment of pain in patients [4]. Nowadays SCS is still used for chronic pain treatment with modified leads, advanced remote pulse generators, and various stimulation parameters/programs such as conventional SCS (i.e., tonic stimulation, at a frequency of 30-80 Hz, 100 to 500 *μ*s of pulse width, and an amplitude above sensory threshold), high-frequency stimulation (at a frequency of 1-10 kHz, with a pulse width at approximately 30 *μ*s, and an amplitude of typically 1 to 5 mA), high-frequency burst stimulation (at a frequency of 40 Hz with 5 closely spaced pulses at 500 Hz per burst), and dorsal root ganglion stimulation [5–7]. Low-frequency SCS has been suggested to be better for treatment of heat hyperalgesia due to C-fiber neuropathy, while high-frequency SCS may be better for modulation of mechanical allodynia due to A-fiber neuropathy [8]. More recently, high-frequency simulation (500 Hz) as compared to low-frequency (5 Hz) and conventional stimulation has been shown to induce a delayed effect on mechanical allodynia in an animal model of painful diabetic polyneuropathy [9]. However, some limitations of SCS have been reported during clinical applications including equipment-associated limitations, such as contamination of implanted leads or pulse generator, the pain caused by the pressure of implanted leads on the nervous system, or discomfort caused by the implanted pulse generator device [10–12]. Current investigations are trying to balance between the advantages and disadvantages of the SCS technique.

Conventional SCS directly stimulates the large diameter nonnociceptive A*β*-fibers in the dorsal column, and then it antidromically inhibits those nociceptive signals which enter the spinal dorsal horn. The electrical pulses generated by the stimulator propagate not only antidromically but also orthodromically along the nonnociceptive nerve fibres. Although it was known that the activation of “Gate control” is attributed to antidromic stimulation, the effects of orthodromic stimulation were largely unknown at that time. In the following two decades, the mechanisms of SCS-induced analgesic effects at the supraspinal level were gradually unraveled. It was found that SCS can modulate pain perception by the activation of some supraspinal pain processing systems such as the thalamic centrum medianum and the pretectal nucleus [13, 14]. Since the discovery of the descending bulbospinal pathways [15], accumulating evidence has shown that the nociceptive-evoked activity in a number of supraspinal areas which are related to pain transmission like the locus coeruleus (LC), the rostral ventromedial medulla (RVM), the reticular formation (RF), and the periaqueductal gray (PAG) can be inhibited by electrical stimulation [16, 17]. From then on, a growing number of studies focused on the alterations in inhibitory neurotransmitters including GABA, serotonin, acetylcholine, opioids, and endocannabinoids in response to SCS, indicating that the “spino-bulbo-spinal” loop was tuned by SCS [16, 18, 19]. Since chronic pain always contains emotional, motivational, and cognitive components which are manifested as mood disorders, the investigation on the influence of SCS on these aspects of pain might facilitate the understanding of the mechanisms of SCS-induced analgesia [20]. While a growing number of studies showed that the motor system and the sympathetic system could be modulated by SCS to improve the locomotor function after SCI and alleviate angina pectoris [21, 22], in this review, we focus on the effects of conventional SCS on neuromodulation of peripheral and spinal cord injury-induced central neuropathic pain.

## 2. Mechanisms of Peripheral and Central Neuropathic Pain

Neuropathic pain (NP) is a complex, heterogeneous disorder that affects approximately 8% of the total adult human population and comes with significant burden for both the patient and the healthcare system [23]. The International Association for the Study of Pain (IASP) defines NP as follows: “pain that arises as a direct consequence of a lesion or disease affecting the somatosensory system” [24]. The origin of NP might be either due to nerve injury of peripheral nerves (peripheral neuropathic pain (PNP)) or due to a central nerve injury (central neuropathic pain (CNP)). PNP is common in CRPS, FBSS, and some diseases resulting in peripheral nerve damage like cancer and diabetes, whereas the CNP usually occurs after stroke, spinal cord injury, or multiple sclerosis [25]. NP is characterized by spontaneous pain (which happens spontaneously without stimuli like burning and tingling etc.), allodynia (response to innocuous stimuli), and hyperalgesia (increased response to noxious stimuli).

During PNP, peripheral tissue injury results in the release of inflammatory mediators/cytokines/chemokines (e.g., PGE2, 5-HT, IL-1*β*, TGF-*β*, and chemokine (C-C motif) ligand 2 (CCL2)) and neurotrophic factors (e.g., nerve growth factor) that sensitize nociceptors, leading to altered expression and activity of ion channels in sensory neurons, consequently reducing the mechanical and thermal threshold of nociceptors (peripheral sensitization) [26]; even light-touch mechanoreceptors (e.g., TrkB^+^ fibers), which do not transduct pain signals in physiological state, start to produce allodynia during PNP [27]. The aberrant excited peripheral neurons release massive amounts of neurotransmitters including glutamate and substance P from the central terminals in the dorsal horn of the spinal cord, and this results in activation of AMPA/NMDA and NK receptors, respectively, inducing long-lasting increased excitability of dorsal horn neurons (a process termed central sensitization) [28]. A recent study demonstrated that increased expression of voltage-gated sodium channels like Na_v_1.7 and Na_v_1.8 in spinal interneurons is also involved in central sensitization [29]. In addition, the inflammatory mediators released from injured neurons can trigger the activation of microglia, astrocytes, oligodendrocytes, mast cells, and T-cells which in turn release more pronociceptive factors (IL-6, IL-1*β*, and TNF-*α*) contributing to the development and maintenance of PNP [30]. Descending inhibition systems are involved in pain modulation during PNP [31]. Due to thoracic spinal cord injury in animal pain models, CNP has “above-level” pain (forelimbs), “at-level” pain (trunk), and “below-level” pain (hindlimbs) [32]. The mechanisms of PNP and CNP have many similarities and some differences. PNP and CNP have in common, as a major similarity, the sensitization phenomenon; however, they differ for the injured location and contributions of sensitization. For example, in CNP, peripheral sensitization is only observed in the “above-level” pain state by “retrograde activation” of peripheral neurons [33], and central sensitization contributes to “at-level” pain and “below-level” pain. Additionally, activated microglia can release PGE2 to modulate the pain processing in dorsal horn neurons in “below-level” pain [34]. The treatment of both PNP and CNP is an unmet need for now since the underlying mechanisms are extremely complicated and have not been fully elucidated.

## 3. Analgesic Mechanisms of Conventional SCS

**Figure 1:**
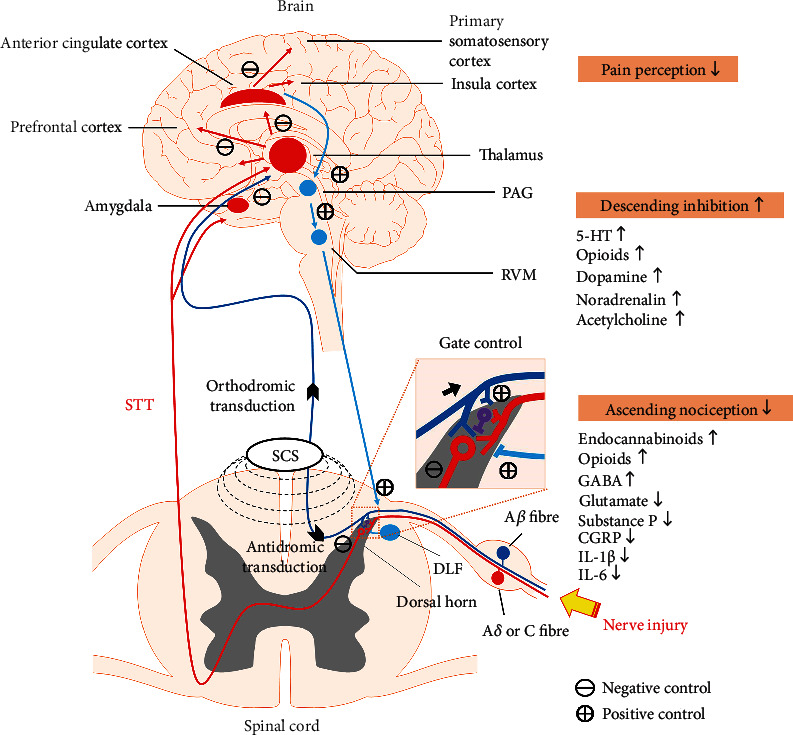
Schematic drawing shows the effects of spinal cord stimulation on nociceptive processing including segmental spinal inhibition, activation of descending inhibitory system, and cortical modulation. SCS: spinal cord stimulation; STT: spinothalamic tract; PAG: periaqueductal gray; RVM: ventrolateral medulla; DLF: dorsolateral funiculus.

The mechanisms of action of SCS were initially modeled with the “Gate control” theory; nonetheless, recent studies have demonstrated the involvement of endocannabinoids, endogenous opioids, and of the descending pain inhibitory systems in the SCS process (Figure 1).

### 3.1. Segmental Inhibition via GABA, Endocannabinoids, and Endogenous Opioids

Segmental inhibition is implemented via activation of GABAergic inhibitory interneurons in the spinal cord and contributes to the SCS-induced analgesia. The earliest evidence of spinal inhibition was discovered by Lidierth and Wall who suggested that dorsal column stimulation might inhibit afferent discharge [35]. However, the best-known mechanism of segmental inhibition is the “Gate control” theory. Based on this theory, the electrical stimulation of large myelinated A*β* fibers, located in the dorsal columns, results in antidromic stimulation of the nociceptive network in the spinal dorsal horn. Indeed, the GABA release from GABAergic inhibitory interneurons in the spinal cord was increased after SCS treatment in animals [18, 36]. The increased GABA activates GABA_A_ receptors on the presynaptic neurons to inhibit the excitatory neurotransmission between glutamatergic nociceptive C-fibers and the wide dynamic range (WDR) neurons in the spinal dorsal horn [36, 37]. A clinical study found that excitability of spinal dorsal horn neurons, particularly WDR neurons, might be inhibited by SCS in chronic neuropathic pain patients [38].

Nevertheless, there is increasing evidence for other mechanisms involved in SCS-induced pain modulation within the spinal “Gate control.” Recently, it has been reported that endocannabinoid activation of cannabinoid receptor 1 (CB_1_R) contributes to long-lasting reversal of neuropathic pain by repetitive SCS to the dorsal columns in rats [39]. It has been demonstrated that SCS can prime the nervous system to evoke more analgesia over time, and this is persistent for several days [39]. Another study consistently reported that blockade of CB_1_R in both excitatory and inhibitory neurons in superficial dorsal horns of the spinal cord attenuated postsynaptic currents caused by electrical stimulation of A*β* fibers [40]. Previous studies carried out in the last 25 years proved that there were two CB_1_R endogenous ligands, N-arachidonoyl-ethanolamine (anandamide) and 2-arachidonoylglycerol (2-AG) [41, 42]. Since their discovery as high (anandamide) and low-to-moderate (2-AG) affinity ligands for CB_1_Rs, it also became clear that the two major endocannabinoids exhibit varying efficacy as CB_1_R agonists [41, 42]. Since CB_1_R is localized preferentially in brain areas involved in pain transmission, such as the cortex, PAG, and in the spinal cord [43, 44], SCS may exert antinociception through activating CB_1_Rs in these areas via both orthodromical and antidromical stimulation.

Moreover, several kinds of other endogenous neurotransmitters including opioids and acetylcholine have been proven to be underlying SCS mechanisms for pain relief [45, 46]. The opioid receptors are involved in the pain relief induced by SCS in a frequency-dependent manner since both 4 Hz and 60 Hz SCS work through opioid receptor mechanisms, with 4 Hz SCS activating *μ*-opioid receptors, while 60 Hz SCS-activated *δ*-opioid receptors [45]. Furthermore, SCS was shown to attenuate peripheral neuropathic pain via activation of the cholinergic system through muscarinic receptor 4, but not through nicotinic receptors in rats [46]. These studies proved that endogenous analgesics or inhibitory mediators are involved in SCS-induced analgesia.

Apart from the alteration of neurotransmitters released in the spinal cord, SCS also reversed the increased pain-related genes which code for proinflammatory cytokines like IL-1 and IL-6 in the dorsal root ganglion in a spared nerve injury model [47], pointing out its role in the peripheral nervous system. Moreover, the release of proinflammatory cytokines by spinal glial cells might be indirectly modulated by SCS. It is known that astrogliosis occurs and microglial GluN2B increases after SCI in rats [48], and the local astroglial scar is proven to hamper the neuroregeneration in the injured spinal cord. Additionally, the proinflammatory cytokines and chemokines released from activated microglia due to SCI contribute to the central sensitization in neuropathic pain. Sato et al. reported that SCS significantly reduced the immunostaining density of marker proteins of astrocytes and microglia bilaterally in rat spinal cord 2 weeks after peripheral nerve injury [49] and SCS attenuated neuropathic pain by suppression of spinal glial activation [50]. The information indicates the possible role of SCS in reducing astrogliosis in spinal-cord-injured rats.

### 3.2. Stimulation-Induced Descending Inhibition

Based on the understanding of descending pain control at the supraspinal level [51, 52] and the orthodromic effect of SCS, SCS might alter the responses of supraspinal systems upon the incoming nociceptive signals in the spinal cord by modulating the balance of descending facilitation and inhibition. A clinical study indirectly proved the role of SCS at the spinal/supraspinal level by increased sensory threshold in both pain areas and nonpain areas of chronic pain patients [53]. It indicates that electrical stimulation may not only activate large myelinated fibers in the dorsal column but also have an influence on the ascending or descending tracts in the ventrolateral column. In agreement, an increasing number of studies demonstrated that descending inhibition was evoked by SCS leading to the release of neurotransmitters including noradrenalin and serotonin which modulate WDR neurons in the spinal cord, and thus playing an important role in the antinociceptive mode of action of SCS.

Moreover, it has been shown that electrical stimulation of the descending fibers originating in certain brainstem areas such as LC and RVM can inhibit the input of nociceptive signals into the brain [54]. Furthermore, electrical stimulation of the A6-A7 nuclei in LC, known to be the source of spinally projecting noradrenergic neurons, inhibits the hypersensitivity in the spinal dorsal horn following noxious challenge [55]. A recent study showed that SCS increased the neuron activity in LC in a peripheral neuropathic pain model but did not change the expression of noradrenaline in the spinal dorsal horn compared to that without SCS [56]. These studies confirmed that the LC neurons are indeed activated by SCS; however, their roles in SCS-induced antinociception needs to be further elucidated.

In contrast to the undefined effects of the noradrenergic system, the role of SCS in the modulation of descending serotoninergic fibers originating from the RVM has been investigated in much more detail. It has been found that the release of 5-HT in the spinal laminae I-II was increased in SCS-treated rats with neuropathic pain [8]. Meanwhile, the immediate early gene c-Fos expression in the RVM was shown to be increased after SCS [9]. Moreover, SCS-induced inhibition of the mechanical hypersensitivity due to peripheral nerve injury could be blocked by intrathecal injection of antagonists of selected serotonergic receptors 5-HT_2A_ and 5-HT_4_ [19]. Recently, it was reported that the dysfunction of descending inhibitory serotonergic neurons in the RVM by microinjection of a GABA_A_ receptor agonist, attenuated the SCS-induced inhibition of nociceptive processing [57]. Although the diverse cell types in the RVM exert different influences at the dorsal horn to facilitate or inhibit nociceptive signal transmission, SCS might tip the balance in favor of inhibition.

PAG is a major nucleus within the midbrain involved in pain inhibition, and it sends its projections via the RVM to the spinal dorsal horn [58]. The descending inhibition of neuronal responses to noxious thermal stimulation is induced by bipolar focal electrical stimulation in PAG [59]. Additionally, 100 Hz SCS with a current of two-thirds of the motor threshold for 30 min and a repeated SCS at 2 hours decrease extracellular concentrations of GABA in ventrolateral PAG in free-moving rats without nerve injury [18]. However, a nonsignificant activation of neurons was noted in the ventrolateral and dorsolateral PAG of spared nerve injured (SNI) rats in response to 100 Hz SCS with a current of 80% of the motor threshold [9]. The discrepancy may occur due to the different use of stimulation parameters as mentioned above and the alteration of nociceptive pathways after SNI. The involvement of PAG in the descending analgesia induced by SCS needs to be confirmed by further studies.

Since the dorsolateral funiculus (DLF) is part of the descending pain inhibitory pathway [60], DLF lesions attenuated the suppressive effect of SCS on thermal hyperalgesia and mechanical allodynia by about 50% in a peripheral neuropathic pain model [61]. Moreover, with the use of two sets of electrodes rostrally at dorsal column nuclei (DCN) and SCS at lower thoracic levels with a DLF lesion at the cervical level in between, the effect of DCN stimulation was equal to that produced by SCS without a DLF lesion, providing further evidence for the involvement of supraspinal control in the mode of action of SCS. With central neuropathic pain due to SCI in patients, the DLF is often also injured and thus the descending inhibitory control is limited. SCS can no longer act via this supraspinal route, and thus, part of the SCS-induced analgesic effect is diminished in the SCI-induced CNP state. In a unilateral dorsal quadrant lesion SCI model in rats, Sun et al. revealed that the DLF lesion not only blocked the majority of the analgesic effect of SCS but also decreased the activation of neural progenitors evoked by SCS 2 weeks after SCI [62]. In conclusion, descending inhibitory systems originating in the brainstem play an essential role in SCS-induced suppression of peripheral and central neuropathic pain.

### 3.3. Modulation of Pain Perception

Dorsal column SCS may have neuromodulatory effects at cortical levels, although understanding the mechanism is extremely complex and far from being completely understood. A recent fMRI study using the partial sciatic nerve ligation-induced PNP model in rats demonstrated that the higher centers of the pain perception system comprising the thalamus, somatosensory cortex, insular cortex, anterior cingulate cortex, limbic network, hippocampus, and nucleus accumbens were tuned by conventional SCS via interactions in multiple pain pathways, and even more brain areas like the raphe nuclei and caudate putamen were activated by an active recharge burst SCS [63]. These massive SCS-evoked brain areas are not only pain related but also cognition/motivation related, suggesting that SCS cannot only reverse the lowered mechanical threshold due to nerve injury but also improve the cognitive-motivational aspects of pain [64]. An experimental study showed that dorsal column stimulation evoked negative responses in somatosensory cortex (SS) I, SSII, and thalamic nuclei in monkeys [65]. In a rat model, expression of c-Fos was increased after SCS treatment in nuclei of the thalamus and forebrain including the insular cortex and amygdala that are involved in the processing of pain [66]. Moreover, a clinical study demonstrated that activation of SSI, SSII, and cingulate regions were detected by functional magnetic resonance imaging (fMRI) in patients with significantly successful pain treatment of SCS [67]. The anterior cingulate cortex was activated by SCS in patients with low limb and back pain to reduce patients' attention to pain [68]. More recently, a cortical function assessment using fMRI elucidated that the cortical connectivity between the somatosensory cortex and limbic areas was decreased by SCS in pain patients with peripheral neuropathic pain (CRPS) [69]. SCS can alleviate not only pain but also anxiety and depression in FBSS patients [70]. Additionally, SCS-induced pain relief is associated with a significant reduction of anxiety, catastrophizing, and disability [71]. It indicated that SCS of the dorsal column may modulate pain perception by reducing negative emotional processing of pain. However, a better understanding of SCS-activated cortical areas/circuits may lead to a more effective and accurate use of SCS for chronic pain relief.

## 4. Clinical Application and Side Effect Concerns

Although the mechanisms underlying SCS-induced pain relief are still not fully understood, this therapy has been used for management of pain for almost half a century [4].

### 4.1. SCS Effects in PNP

With a growing number of clinical trials, it is now obvious that its efficiency varies with the clinical indication tested like CRPS, FBSS, diabetic neuropathy, ischaemic pain, and postherpetic neuralgia [72–77].

The most optimal effectivity of SCS-induced pain relief was reported in a randomized trial of CRPS type 1 patients [73]. This study reported 14 (58%) out of 24 patients implanted with SCS devices (delivering conventional stimulation at 85 Hz) together with physical therapy showed a remarkable and significant reduction (24%) of pain score (visual analogue scale (VAS)) as compared to those patients treated with physical therapy only. Another indication of SCS for pain treatment is FBSS [75, 78, 79]. A randomized controlled trial of 50 patients with SCS-device implantation (offering conventional stimulation at 49 Hz) illustrated that 48% of the patients achieved pain relief in legs in the intention-to-treat at 6 months, which was significantly more efficient than that (9%) of the conventional medical management group [75]. To improve the responsive rate of SCS, more stimulation programs have been developed recently. For example, high-frequency stimulation (10 kHz) or burst stimulation reduced VAS in 14 out of 16 refractory FBSS patients, which means the responsive rate can reach to 88% [80]. For FBSS patients whose back pain reduction was less than 50% after trial SCS, additional subcutaneous stimulation to SCS significantly suppressed their back pain compared to those controls with subcutaneous lead implantation but switched off [81]. Moreover, a recent study on the effectiveness of SCS in patients with painful diabetic neuropathy (PDNP) provided evidence that SCS over a six-month period could significantly reduce the pain scores during both daytime and nighttime, compared to most efficient pain relief induced by medical treatment [76]. Another randomized clinical trial showed that the average VAS score of PDNP patients was significantly reduced by six months of treatment of SCS from 73 to 31 (*n* = 40), whereas there was no change in the control group (VAS score = 67; *n* = 20) [82]. Additionally, peripheral ischaemic pain due to diabetes and ischaemic ulceration in 25 patients was significantly attenuated by SCS during an 18-month follow-up period, compared to the control group [72]. Furthermore, an investigation over 29 months (median) found that SCS provided a long-term pain relief in 23 out of 28 patients suffering refractory postherpetic neuralgia for more than 2 years [74]. A recent clinical study reported that 60-70% of patients diagnosed as CRPS, and postherpetic neuralgia had a significant lower VAS score after 12-month SCS treatment, compared to baseline [77]. Moreover, as we have known that not all the patients have responses to SCS, about 2/3 of CRPS patients do not respond to SCS [37], and PDNP patients are susceptible to infection; therefore, a trial period of SCS is required before permanent device implantation.

Taken together, the European Federation of Neurological Societies (EFNS) and the IASP Special Interest Group on Neuropathic Pain (NeuPSIG) gave SCS a weak recommendation for CRPS and FBSS [83, 84]. The European Academy of Neurology also weakly recommended SCS for diabetic painful neuropathy [85]. However, the National Institution of Health and Care Excellence (NICE) published a guide for UK and most of Europe and recommended SCS as an option for treating chronic pain of neuropathic and ischaemic origin [86].

### 4.2. SCS Effects in CNP

A literature review about the clinical practice of SCS for treatment of SCI-induced neuropathic pain by searching MEDLINE and EMBASE databases showed that 9 out of 22 case studies reported more than 50% pain relief was achieved by conventional stimulation, 3 out of 22 reported 30-80% pain reduction was obtained by high-frequency stimulation, and 1 out of 22 reported 30% pain score was reduced by burst stimulation. Although the quality of these case studies was low, they support the pain-relief efficacy and safety of SCS in SCI and point out the possibility of clinical practice of SCS for the management of NP after SCI [87]. Together with the preclinical study of SCS in the SCI pain model, which reported that a low-frequency (10-25 Hz) stimulation was associated with neural progenitor activation in the spinal cord and led to long-lasting analgesia [62], conventional SCS with lower frequency might be more efficient in SCI patients. Moreover, the timing of SCS during development of NP is also important for increasing the responsive rate. Early SCS (24 h after nerve injury) increased the number of responders and the duration of analgesic effect than late SCS (16 d after injury) [88], suggesting that SCS should be practiced as early as possible (i.e., the sooner, the better). On the other hand, as an invasive treatment, the risk of complications of SCS due to lead infection and dislocation is unneglectable. Evidence showed that 32% of patients developed device-related complications after 12-month implantation [75]. The adverse events in cervical SCS treatment for chronic pain were hardware malfunction (17.8%), lead migration (13.9%), and lead breakage (6.7%) [10]. A rare complication of SCS that can occur using leads placed via open surgical approach is spinal and radicular compression symptoms caused by the growth of fibrotic epidural mass at the level of the lead [11]. Spinal hematoma due to paddle leads for SCS was also found with 0.63% incidence (18/2868 patients) within 30 days following operation [12]. Therefore, the disadvantages of SCS along with the extent of damage in the individual spinal cord-injured patients should be considered carefully before SCS for treatment of CNP.

To date, despite recent progress and insights into mechanisms involved in SCS-induced pain relief in CNP, this therapy is not yet approved for use in clinic and SCI patients. Future studies should be designed to generate robust evidence about the benefits of SCS (including pain relief, function, and quality of life) in the SCI-induced CNP condition.

## 5. Summary

The analgesic mechanisms of SCS are gradually unveiled with the involvement of the “Gate control” theory, segmental inhibition, the descending inhibitory system, and cortical modulation. However, SCS is still an evidence-based interventional therapy for use in humans. It is recommended for selected indications related to PNP-like patients who experience refractory pain including CRPS and FBSS. Since the majority of mechanism studies on CNP and SCS are based on the experimental contusion/transection-induced SCI pain models, SCS may be considered as a potential therapy to treat trauma-induced CNP in patients, whereas SCI due to degenerative pathologies and somatic infection is a contraindication of SCS.

## Data Availability

No data were used to support this study.
